# Environmental Enrichment Rescues Social Behavioral Deficits and Synaptic Abnormalities in *Pten* Haploinsufficient Mice

**DOI:** 10.3390/genes12091366

**Published:** 2021-08-30

**Authors:** Amy E. Clipperton-Allen, Angela Zhang, Ori S. Cohen, Damon Theron Page

**Affiliations:** Department of Neuroscience, The Scripps Research Institute, Jupiter, FL 33458, USA; aallen@scripps.edu (A.E.C.-A.); azhang2@uw.edu (A.Z.); oricohen@hotmail.com (O.S.C.)

**Keywords:** environmental enrichment, synaptic plasticity, *Pten*, autism, neurodevelopment

## Abstract

*Pten* germline haploinsufficient (*Pten*^+/−^) mice, which model macrocephaly/autism syndrome, show social and repetitive behavior deficits, early brain overgrowth, and cortical–subcortical hyperconnectivity. Previous work indicated that altered neuronal connectivity may be a substrate for behavioral deficits. We hypothesized that exposing *Pten*^+/−^ mice to environmental enrichment after brain overgrowth has occurred may facilitate adaptation to abnormal “hard-wired” connectivity through enhancing synaptic plasticity. Thus, we reared *Pten*^+/−^ mice and their wild-type littermates from weaning under either standard (4–5 mice per standard-sized cage, containing only bedding and nestlet) or enriched (9–10 mice per large-sized cage, containing objects for exploration and a running wheel, plus bedding and nestlet) conditions. Adult mice were tested on social and non-social assays in which *Pten*^+/−^ mice display deficits. Environmental enrichment rescued sex-specific deficits in social behavior in *Pten*^+/−^ mice and partially rescued increased repetitive behavior in *Pten*^+/−^ males. We found that *Pten*^+/−^ mice show increased excitatory and decreased inhibitory pre-synaptic proteins; this phenotype was also rescued by environmental enrichment. Together, our results indicate that environmental enrichment can rescue social behavioral deficits in *Pten*^+/−^ mice, possibly through normalizing the excitatory synaptic protein abundance.

## 1. Introduction

A subset of individuals with autism spectrum disorder (ASD) display overgrowth of the head (macrocephaly) and brain (megalencephaly). Up to 25% of these cases are classified as macrocephaly/autism syndrome (OMIM #605309), which is caused by mutations in the *Phosphatase and tensin homolog* (*PTEN*) gene [1,2,3,4,5,6,7]. These are typically germline, heterozygous, loss-of-function mutations [3,8,9,10]. *PTEN* codes for a negative regulator of the PI3K-Akt-mTOR pathway, which is also the target of other ASD risk factors, including *TSC1/2*, *NF1*, and *FMR1* [11,12,13].

We have previously examined macrocephaly/autism syndrome using mice with germline *Pten* haploinsufficiency (*Pten*^+/−^), which are a model for the *PTEN* mutations found in humans with macrocephaly/autism syndrome. These mice have abnormal social and non-social behavior [14,15,16,17] on tests related to the core symptoms of ASD (social behavior and communication deficits, and restricted, repetitive behavior and interests [18]). Additionally, these mice exhibit cortical–subcortical hyperconnectivity, and brain overgrowth that is present from birth and persists into adulthood; this overgrowth is primarily due to hyperplasia, but also to hypertrophy in a subset of neuronal cell types [14,17,19,20,21,22]. 

*Pten*^+/−^ mice also show sexual dimorphism on almost all behavioral assays (see [16] for a review). Sex differences are well known in the ASD population, with a male-to-female ratio of approximately 3–3.5:1 [23]. While diagnostic biases may account for some of these differences [24,25], they are unlikely to explain the entirety of this disproportionate impact on males. Proposed explanations for the male predominance include the extreme male brain theory [26] and the female protective effect theory [27,28].

One of the most reliable treatments for ASD is early behavioral intervention [29,30]. However, the neurobiological underpinnings of such a treatment are still unclear. Environmental enrichment (EE) in animals can model these therapies. In addition to rescuing behavioral deficits in other mouse models of ASD risk factors in the PI3K-Akt-mTOR pathway (e.g., [31,32]), EE has been shown to have numerous molecular, cellular, neuroanatomical, and other behavioral effects (reviewed in [33,34]). Of particular interest, EE can increase synaptic plasticity, strength, and long-term potentiation (LTP) and depression (LTD) by altering the expression of genes associated with synaptic function and cellular plasticity, increasing the expression of synaptic proteins, synaptogenesis, and altering NMDA and AMPA receptor subunits (e.g., [34,35,36,37]). 

**Figure 1:**
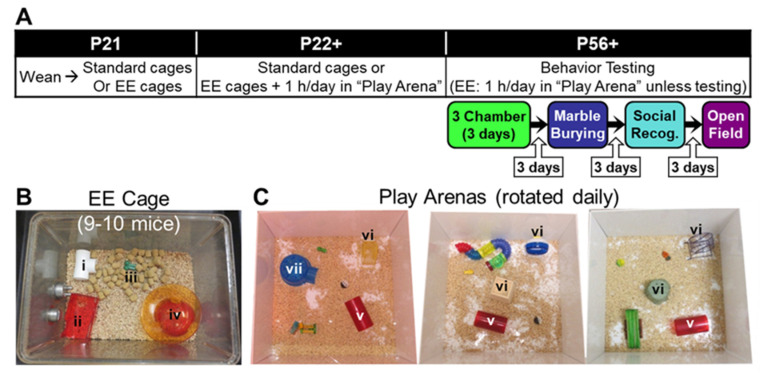
Experimental design. (**A**) Timeline from beginning of environmental enrichment (EE) to end of testing. (**B**) EE cage. (**C**) Play arenas with the three different toy groups. EE mice received 1 h in the play arena on every day they were not behaviorally tested from weaning. i, White t-shaped tube; ii, red two-level house; iii, green chew toy; iv, igloo-style hut with saucer running wheel; v, large red tube; vi, running wheel; vii, shelter.

We hypothesized that manipulating plasticity in *Pten*^+/−^ mice after the establishment of brain overgrowth, but during postnatal development, may facilitate adaptation to the abnormal “hard-wired” connectivity. Thus, *Pten*^+/−^ mice and their wild-type (*Pten*^+/+^) littermates were weaned into same-sex standard or EE cages, where they remained for the duration of the experiment. All cages contained a mix of both genotypes. EE cages were larger and contained more mice (social enrichment) and more toys (including a running wheel); mice in these cages also received 1 h each day in a play arena, a large open field containing a set of toys that was rotated daily, except on days that they received a behavioral test (see Figure 1). Starting in early adulthood, mice were tested on ASD-like behavioral assays that had previously shown a phenotype in untreated *Pten*^+/−^ mice, including the three-chamber social approach, social recognition, and marble burying, as well as the open field test as a locomotor and anxiety control behavior. Following behavioral testing, we explored whether changes in excitatory or inhibitory pre- or post-synaptic proteins could account for improved behavioral outcomes. 

## 2. Materials and Methods

### 2.1. Mice

All mice used have been described previously. Mice of the *B6.129-Pten^tm1Rps^* line (RRID: MGI:2179044 [38]) were obtained from the repository at the National Cancer Institute at Frederick, where they were already backcrossed onto a congenic C57BL/6J background by the donating investigator. The line has been maintained by backcrossing to C57BL/6J mice (RRID: IMSR_JAX:000664; strain #000664, The Jackson Laboratory, Bar Harbor, ME, USA) for more than 10 generations. Mice used in this study were generated by crossing *Pten^tm1Rps/+^* (*Pten*^+/−^) male mice with wild-type (*Pten*^+/+^) females. After weaning, mice were held on ventilated racks (Allentown Inc., Allenton, NJ, USA) as described below and provided with food (Teklad Global 18% Protein Extruded Rodent Diet 2920X, Harlan Laboratories, Indianapolis, IN, USA) and water ad libitum.

All research was approved by The Scripps Research Institute’s Institutional Animal Care and Use Committee and conducted in accordance with National Institutes of Health and Association for Assessment and Accreditation of Laboratory Animal Care International (AAALAC) guidelines.

### 2.2. Environmental Enrichment

Mice were weaned into either standard (Std-*Pten*^+/+^ and Std-*Pten*^+/−^) or EE (EE-*Pten*^+/+^ and EE-*Pten*^+/−^) same-sex cages at P20-22. Litters were combined to reach the necessary number and genotype composition (approximately 50% each of *Pten*^+/+^ and *Pten*^+/−^ mice) for each cage. Standard housing consisted of 4–5 mice in a clear polyethylene cage (standard mouse size, 19.1 × 29.2 × 12.7 cm; Allentown Inc., Allentown, NJ, USA) with ¼” corncob bedding; a single nestlet was also provided. EE housing (see Figure 1B) consisted of 9–10 mice in a larger clear polyethylene cage (standard rat size, 39.4 × 28.6 × 19.1 cm; Allentown Inc.), with ¼” corncob bedding, and containing two nestlets, a white t-shaped tube (i in Figure 1B), a red two-level house (ii in Figure 1B), a green chew toy (iii in Figure 1B), and an igloo-style hut with a “saucer” running wheel (iv in Figure 1B). Additionally, EE mice received 1 h each day in a large white open “play arena” (60 × 60 × 30 cm) containing a selection of toys that were rotated daily (see Figure 1C), but which always included a large red tube (v in Figure 1C), a vertical running wheel (vi in Figure 1C), and some type of shelter (vii in Figure 1C). Play arena time occurred every day for the first 35 days (from weaning until the beginning of testing), and every subsequent day on which mice were not given a behavioral test (see Figure 1A). All play arena time took place during the dark phase of the light/dark cycle under red light.

### 2.3. Behavioral Assays

#### 2.3.1. General Procedures

All behavior tests were performed on both male and female mice, and the sexes were analyzed separately, as sexual dimorphism is common in mice with *Pten* mutations (see [16] for a review). Behavior tests were performed during the dark (active) phase of the reversed light/dark cycle under red light, unless otherwise specified, with at least 3 days between assays. The battery of tests was performed in the same order by all mice (three-chamber social approach, marble burying, social recognition, open field test; see Figure 1A) and carefully designed to minimize carryover effects. Mice were moved into the testing area at least 1 h prior to testing. Apparatuses were cleaned with 70% ethanol (EtOH; Sigma-Aldrich, St. Louis, MO, USA), 1% Micro-90 (International Products Corporation, Burlington, NJ, USA), and/or quatricide (2 oz/gallon; Pharmacal Research Laboratories, Inc., Waterbury, CT, USA), unless otherwise stated. Manual scoring (marble burying, social recognition (with a stopwatch)) was performed by a trained observer blind to sex and genotype, and automatic scoring (three-chamber social approach, open field test) was performed using the Ethovision XT video tracking system (RRID:SCR_000441; Noldus Leesburg, VA, USA)). Details of these paradigms are listed below.

#### 2.3.2. Three-Chamber Social Approach

Mice were tested under dim white light as previously described [17,39,40]. Briefly, following 5 min of acclimation to an empty acrylic arena partitioned into three chambers with a white floor and black walls, mice were given a 10 min social approach trial, in which they could choose between spending time in the “empty tube” chamber (containing an empty acrylic tube with holes in the bottom third of the tube) or the “mouse + tube” chamber (containing an identical tube with a same-sex stimulus mouse in it). Stimulus location was counterbalanced across mice. Social stimulus animals were adult (approximately 7-month-old) same-sex wild-type (WT) mice on a C57BL/6J background and were from different breeding cages than the test mice. Trials were automatically scored for chamber time, distance from each tube, and distance traveled.

#### 2.3.3. Social Recognition

Social recognition was measured using a habituation/dishabituation procedure as previously described [14,39]. Briefly, test mice were individually placed in clean home cage-like environments with an acrylic tube containing holes in the bottom third of the tube for 2 h [41]. Following this acclimation, mice received five 5 min presentations of same-sex juvenile (P21–28) conspecifics with 15 min intertrial intervals; the same mouse was presented in the first four trials (H1–H4), with a novel juvenile in the fifth (“test”) trial. Stimuli were WT mice on a C57BL/6J background, from different breeding cages than the test mice and the stimulus animals used for the three-chamber social approach test above. The duration of investigation was manually scored from video, with mice required to spend at least 10 s investigating the stimulus during the first habituation (which all mice did). 

#### 2.3.4. Marble Burying

Mice were individually placed into a clean home cage-like environment containing ¼ inch corncob bedding to a depth of 5 cm, with 20 black marbles (msc916bl, GlassMarbles.com) arranged in a 4 × 5 matrix, as previously described [14,39]. After 30 min undisturbed, the number of marbles that were at least 2/3 buried were counted [42].

#### 2.3.5. Open Field Test

To assess potential effects of EE on locomotion and anxiety, mice underwent the open field test (OFT) as previously described [14,39]. Briefly, each mouse was placed in the center of an open field arena under ~240 lux of white light and given 5 min to explore. Times spent in the center and in thigmotaxis (occupying the corners and sides of the arena) were automatically recorded as anxiety measures, and the total distance traveled was measured to assess locomotion.

### 2.4. Post-Mortem Analyses

#### 2.4.1. Brain and Body Mass

Mice were sacrificed following the completion of behavioral testing. This was conducted in one of two ways: (1) transcardial perfusion with PBS (Life Technologies, Carlsbad, CA, USA) followed by 4% paraformaldehyde (PFA; Sigma-Aldrich), post-fixation in 4% PFA overnight, and then storage in 20% sucrose until embedded in Tissue-Tek OCT compound (VWR, West Chester, PA, USA) and frozen at −80 °C, as previously described [20,39]; (2) cervical dislocation and decapitation, followed by rapid extraction and flash freezing of the brain tissue in 2-methylbutane (Thermo Fisher Scientific, Asheville, NC, USA) placed in a dry ice and 70% EtOH bath, and storage at −80 °C until use for analysis below. In all cases, the brain and body masses of the mice were recorded.

#### 2.4.2. Synaptosomal Preparation and Western Blot Analysis

The anterior third of the cortex, excluding the olfactory bulbs, was dissected using a brain matrix from frozen brains of female Std-*Pten*^+/+^, EE-*Pten*^+/+^, Std-*Pten*^+/−^, and EE-*Pten*^+/−^ mice; some standard-housed mice had not been tested on behavior prior to collection. Protein was extracted using the Syn-PER^TM^ Synaptic Protein Extraction Reagent (Thermo Fisher Scientific) following the manufacturer’s protocol, with phosphatase (phosphatase inhibitor cocktails 2 and 3, Sigma-Aldrich) and protease (EDTA-free cOmplete Ultra, Sigma-Aldrich) inhibitors included in the lysis buffer. Samples were diluted for equal amounts of protein per lane based on concentrations found using the Pierce BCA Protein Assay Kit (Thermo Fisher Scientific). Proteins were electrophoresed onto NuPAGE 4–12% Bis-Tris Gels (Novex, Life Technologies) and then transferred to polyvinylidene difluoride membranes (Thermo Fisher Scientific). Primary antibodies were vGluT1 (1:500; RRID:AB_2797887; #12331, Cell Signaling Technology, Danvers, MA, USA), vGAT (1:1000; RRID:AB_2189938; #131013, Synaptic Systems, Goettingen, Germany), PSD-95 (1:250; RRID:AB_2533914; #51-6900, Thermo Fisher Scientific), gephyrin (1:1000; RRID:AB_2798443; #14304, Cell Signaling Technology), and β-actin (concentration 1:2000; RRID:AB_2223172; #4970, Cell Signaling Technology). The secondary antibody used was peroxidase-conjugated anti-rabbit IgG (1:5000; RRID:AB_10015282; Jackson ImmunoResearch Laboratories, Inc., West Grove, PA, USA), and proteins were visualized with chemiluminescence enhanced by the WesternBright Quantum Kit (Advansta, San Jose, CA, USA). Quantification was performed using the blot tool in ImageJ to measure relative abundance, and proteins were normalized to β-actin.

### 2.5. Statistical Analysis

Behavioral results from male and female mice were analyzed separately, as we have previously shown that sexual dimorphism in behavioral phenotypes is extremely common in the *Pten*^+/−^ line (as it is in most *Pten* mutant mice; see [16] for a review).

To compare effects of genotype and condition on dependent variables, we used two-way between-subjects analyses of variance (ANOVAs), as well as planned comparisons (effect of genotype for each condition and effect of condition for each genotype). Specifically, these tests were performed for distance traveled (three-chamber social approach, OFT); H1, test, habituation, and dishabituation scores (social recognition); percent of marbles ⅔+ buried (marble burying); % center time (OFT); brain mass, body mass, and brain as a percent of body mass; and vGluT1/β-actin, vGAT/β-actin, PSD-95/β-actin, and gephyrin/β-actin (Synaptosomal Western Blots). Additional statistical tests included three-way mixed-model ANOVAs (three-chamber social approach: genotype × condition × chamber on % chamber time; social recognition: genotype × condition × trial on investigation time; OFT: genotype × condition × thigmotaxis/center on duration). Planned comparisons also assessed effects of chamber and tube distance (three-chamber social approach: paired *t*-tests comparing mouse + tube and empty tube), trial (social recognition: one-way within-subjects ANOVA for H1–H4), habituation and dishabituation scores (social recognition: one-sample *t*-test vs. 0), and center vs. thigmotaxis time (OFT: paired *t*-tests) separately for each group (i.e., Std-*Pten*^+/+^, EE-*Pten*^+/+^, Std-*Pten*^+/−^, and EE-*Pten*^+/−^). Sidak or Tukey post hoc tests were used when interactions were significant.

All statistical analyses were performed with PASW 18 (IBM Corporation, Armonk, NY, USA), with significance set at *p* < 0.05. Complete statistical results are presented in Supplemental Tables S1–S3 and thus are not included in detail in the text.

## 3. Results

### 3.1. Environmental Enrichment Rescues Social Behavioral Deficits in Pten^+/−^ Mice

We have previously identified social deficits in both sexes of *Pten*^+/−^ mice, but of different types. Female *Pten*^+/−^ mice fail to show a social preference in the three-chamber social approach assay, suggesting decreased social interest, as well as decreased investigation during the first trial of the habituation/dishabituation social recognition test [14,17,21,43]. Male *Pten*^+/−^ mice have occasionally failed to show a social preference in the three-chamber social approach paradigm (e.g., [14]) but typically perform normally on this task (e.g., [17]). However, they have shown impaired social recognition in multiple paradigms (e.g., [14,17]. Thus, we tested mice of both sexes on both social interest (three-chamber social approach) and social recognition (habituation/dishabituation) assays following rearing in standard or EE conditions (Figure 1).

**Figure 2:**
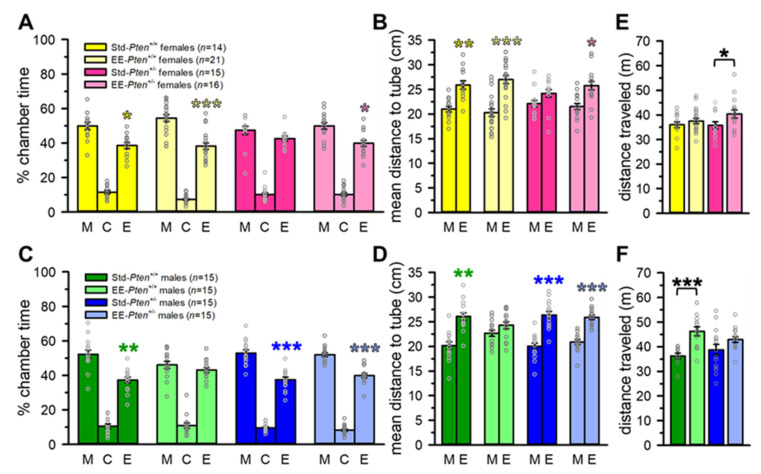
Environmental enrichment rescued social approach deficits in *Pten*^+/−^ females. (**A**,**B**) All females except for Std-*Pten*^+/−^ mice showed a preference for the social chamber (**A**) and were, on average, closer to the tube with a social stimulus (**B**). (**C**,**D**) All males except for EE-*Pten*^+/+^ mice showed a preference for the social chamber (**C**) and were, on average, closer to the tube with a social stimulus (**D**). (**E**,**F**) EE increased the distance traveled by *Pten*^+/−^ females (**E**) and *Pten*^+/+^ males (**F**). Mean ± SEM. Black symbols, difference between conditions. Colored symbols, within-group differences. *** *p* < 0.001, ** *p* < 0.01, * *p* < 0.05. M, mouse + tube chamber; C, center chamber; E, empty tube chamber; Std, standard housing; EE, environmental enrichment.

We replicated our previous finding of impaired social preference in female *Pten*^+/−^ mice. Std-*Pten*^+/+^ females spent significantly more time in the mouse + tube than the empty tube chamber (see Figure 2A) and were significantly closer, on average, to the tube containing a stimulus mouse than the empty tube (see Figure 2B), indicating a significant social preference, while female Std-*Pten*^+/−^ mice did not show these behavoirs (see Figure 2A,B, Supplemental Table S1). However, when raised with EE, both EE-*Pten*^+/+^ and EE-*Pten*^+/−^ females showed significant social preferences (see Figure 2A,B, Supplemental Table S1). Standard males of both genotypes, and EE-*Pten*^+/−^ males, also showed social preferences, although EE-*Pten*^+/+^ male mice did not (see Figure 2C,D, Supplemental Table S1). While there were no genotype differences in distance traveled during the trial in standard mice, EE did have a significant main effect; specifically, EE-*Pten*^+/−^ females and EE-*Pten*^+/+^ males traveled farther than standard mice of the same genotype (see Figure 2E,F, Supplemental Table S1). 

**Figure 3:**
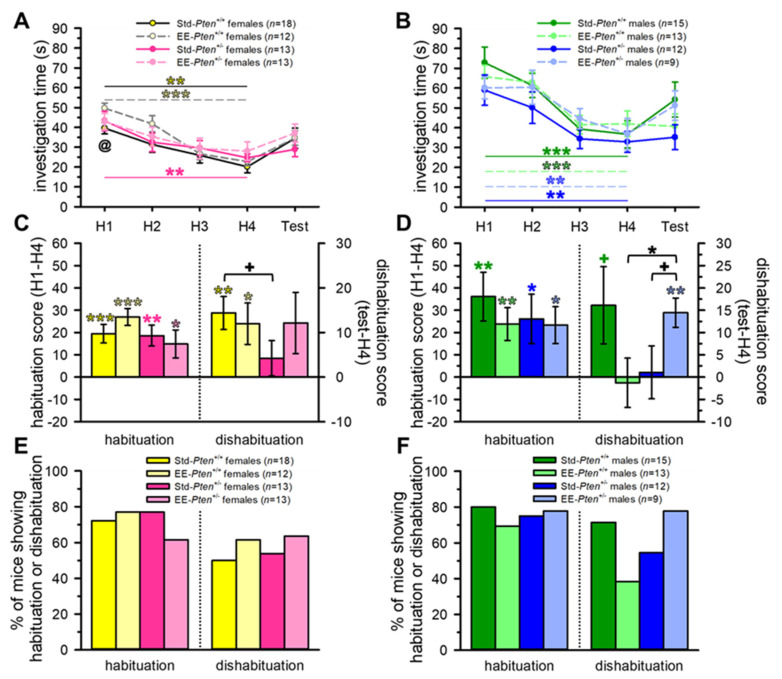
Environmental enrichment rescues social recognition deficits in *Pten*^+/−^ male mice. (**A**,**B**) All female (**A**) and male (**B**) groups except EE-*Pten*^+/−^ females showed a significant reduction in investigation across trials from H1 to H4. EE-*Pten*^+/−^ females also investigated less than female EE-*Pten*^+/+^ controls (**A**). (**C**,**D**) Female (**C**) and male (**D**) mice in all groups habituated. Std-*Pten*^+/+^ and EE-*Pten*^+/+^, but not Std-*Pten*^+/−^ or EE-*Pten*^+/−^, females dishabituated, with a trend to a lower dishabituation score in Std-*Pten*^+/−^ than Std-*Pten*^+/+^ females (**C**). Std-*Pten*^+/+^, but not EE-*Pten*^+/+^, males and EE-*Pten*^+/−^, but not Std-*Pten*^+/−^, males dishabituated, with a genotype difference in EE mice, and an effect of enrichment on *Pten*^+/−^ mice (**D**). (**E**,**F**) Percent of female (**E**) and male (**F**) mice tested that showed habituation or dishabituation. Mean ± SEM. Black symbols, difference between genotypes or conditions. Colored symbols, effect of trial (one-way ANOVA; (**A**,**B**)) or difference from 0 (one-sample *t*-test; (**C**,**D**)). ^@^ Difference between EE-*Pten*^+/+^ and EE-*Pten*^+/−^ females, *p* < 0.05. *** *p* < 0.001, ** *p* < 0.01, * *p* < 0.05, ^+^ *p* < 0.10. H1–H4, habituation trials 1–4; Std, standard housing; EE, environmental enrichment.

Consistent with our prior findings [14], Std-*Pten*^+/+^ males, and standard females of both genotypes, showed normal habituation in the social recognition test, both showing a significant decrease in investigation across habituation trials (main effect of trial) and a significant habituation score (H1 investigation–H4 investigation; see Figure 3A–F, Supplemental Table S1). Std-*Pten*^+/−^ males did show significant habituation (decrease in investigation across habituation trials, habituation score), but no significant dishabituation (test investigation–H4 investigation; Figure 3B,D,F, Supplemental Table S1). EE rescued this deficit in social recognition, with significant dishabituation scores in the EE-*Pten*^+/−^ males and a trend to an increased dishabituation score when compared to Std-*Pten*^+/−^ male mice (see Figure 3B,D,F, Supplemental Table S1). However, EE had the opposite effect on *Pten*^+/+^ control males, who failed to show significant dishabituation, resulting in a lower dishabituation score than EE-*Pten*^+/−^ males (see Figure 3D,F, Supplemental Table S1).

Interestingly, in this cohort, Std-*Pten*^+/−^ female mice had a lower dishabituation score than Std-*Pten*^+/+^ females and, as with Std-*Pten*^+/−^ males, failed to show significant dishabituation when presented with a novel juvenile stimulus in the test trial (see Figure 3A,C, Supplemental Table S1). EE females showed similar patterns to the standard mice, with both genotypes showing significant habituation scores, and only EE-*Pten*^+/+^ females showing significant dishabituation (see Figure 3C). However, female EE-*Pten*^+/−^ mice showed decreased social interest (less investigation than EE-*Pten*^+/+^ females in H1) and no significant main effect of trial (H1 to H4; see Figure 3A, Supplemental Table S1). 

Taken together, these data suggest that being raised with EE can rescue the deficits in social approach in female and social recognition in male *Pten*^+/−^ mice. Interestingly, EE appears to alter normal patterns of social behavior in male *Pten*^+/+^ (control) mice, resulting in decreased social interest and impaired social recognition.

### 3.2. Environmental Enrichment Partially Rescues ASD-Relevant Repetitive Behavior in Pten^+/−^ Male Mice

**Figure 4:**
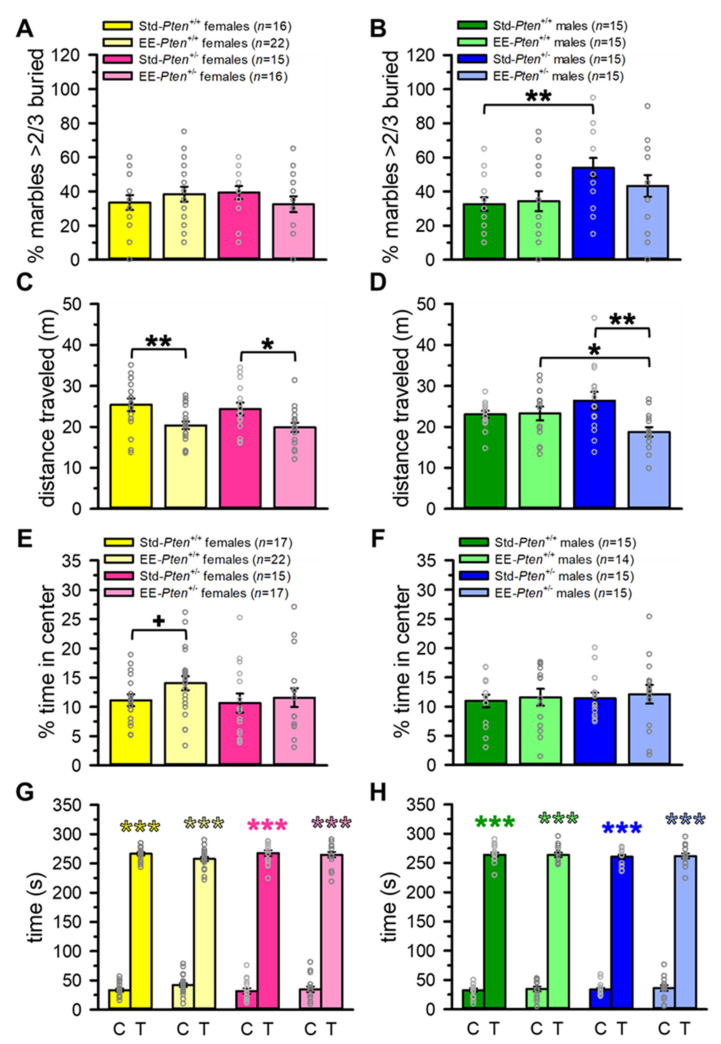
Environmental enrichment rescues *Pten*^+/−^ male repetitive behavior phenotype. (**A**,**B**) Male Std-*Pten*^+/−^ mice buried more marbles than Std-*Pten*^+/+^ males, but no genotype difference was found in EE males (**B**). Females showed no difference between any groups or conditions (**A**). (**C**,**D**) EE decreased distance traveled in both genotypes of females (**C**) and *Pten*^+/−^ males (**D**), and EE-*Pten*^+/+^ males traveled farther than EE-*Pten*^+/−^ males (**D**). (**E**–**H**) Enrichment had a trend to decreasing anxiety in *Pten*^+/+^ females (**E**). No other genotype or condition differences were found in anxiety (center time) in females (**E**) or males (**F**), and all female (**G**) and male (**H**) groups spent significantly longer in thigmotaxis than the center of the arena. Mean ± SEM. Black symbols, difference between genotypes or conditions. Colored symbols, difference between center and thigmotaxis time (paired-sample *t*-test). *** *p* < 0.001, ** *p* < 0.01, * *p* < 0.05, ^+^ *p* < 0.10. C, center of arena; T, thigmotaxis; Std, standard housing; EE, environmental enrichment.

We have previously shown that *Pten*^+/−^ male mice exhibit increased repetitive digging behavior, both using the marble burying test and during a free social interaction [14,15]. Thus, we wanted to determine if this behavior could also be rescued by EE. We found that, as expected based on our previous results [14], there were no differences between any female groups (see Figure 4A, Supplemental Table S2). We also replicated our previous finding, as Std-*Pten*^+/−^ males buried significantly more marbles than Std-*Pten*^+/+^ controls; this was partially rescued by EE, as there were no significant differences between either EE-*Pten*^+/+^ and EE-*Pten*^+/−^ mice or Std-*Pten*^+/−^ and EE-*Pten*^+/−^ mice (see Figure 4B, Supplemental Table S2). 

As we had observed some differences in locomotion on the 3-chamber social approach assay, mice were tested for locomotor and anxiety behavior in the OFT. Interestingly, while EE again altered the distance traveled during the trial, it was in the opposite direction from the 3-chamber social approach test. In both *Pten*^+/+^ and *Pten*^+/−^ females, as well as *Pten*^+/−^ males, distance traveled was decreased in the EE mice in comparison to standard-housed mice of the same genotype (see Figure 4C,D, Supplemental Table S2). Additionally, EE-*Pten*^+/−^ males traveled a shorter distance than their EE-*Pten*^+/+^ cagemates (see Figure 4D, Supplemental Table S2). While we failed to replicate our previous finding of decreased anxiety in Std-*Pten*^+/−^ males [14], we did see a trend to a decrease in anxiety in EE-*Pten*^+/+^ females relative to Std-*Pten*^+/+^ females, and all groups showed a significant preference for thigmotaxis over center time (see Figure 4E–H, Supplemental Table S2). 

These data confirmed that in standard conditions, male *Pten*^+/−^ mice show increased marble burying, and that EE partially rescues this phenotype. In the OFT, like in the 3-chamber social approach assay above, EE altered locomotor behavior, but in the opposite direction, decreasing distance traveled in both genotypes of females and *Pten*^+/−^ males. Little to no effects of genotype or condition were found for marble burying in females, or for anxiety in either sex in the OFT.

### 3.3. Environmental Enrichment Rescues Pre-Synaptic Protein Levels, but Does Not Modify Brain Overgrowth in Pten^+/−^ Mice

**Figure 5:**
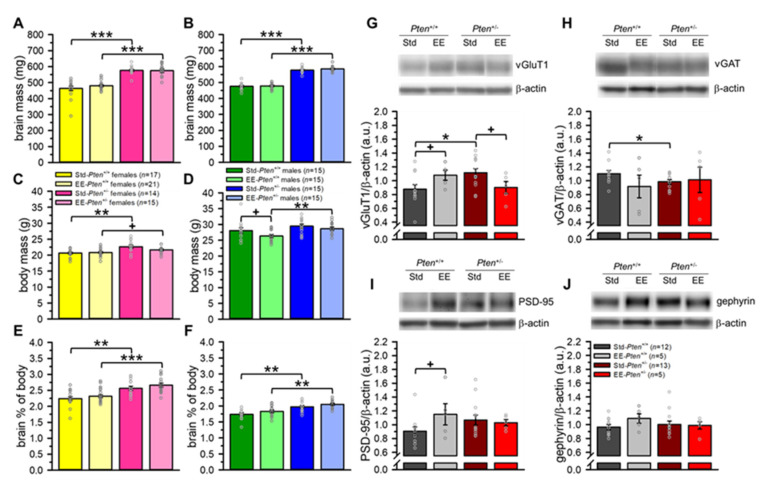
Environmental enrichment has no effect on brain overgrowth, but rescues altered E/I balance in *Pten*^+/−^ mice. (**A**,**B**) Both Std-*Pten*^+/−^ and EE-*Pten*^+/−^ females (**A**) and males (**B**) had increased absolute brain mass relative to *Pten*^+/+^ mice in the same conditions. (**C**,**D**) Body mass in Std-*Pten*^+/+^ and EE-*Pten*^+/+^ females was lighter than Std-*Pten*^+/−^ and EE-*Pten*^+/−^ females, respectively (**C**), and EE-*Pten*^+/+^ male mice were lighter than both EE-*Pten*^+/−^ males and Std-*Pten*^+/+^ males (**D**). (**E**,**F**) Both Std-*Pten*^+/−^ and EE-*Pten*^+/−^ females (**E**) and males (**F**) show increased brain mass relative to body mass (brain mass/body mass × 100). (**G**,**H**) Synaptosomal levels of excitatory (**G**) and inhibitory (**H**) pre-synaptic proteins in the frontal cerebral cortex were altered by genotype and condition. Excitatory pre-synaptic protein vGluT1 was elevated in Std-*Pten*^+/−^ mice relative to Std-*Pten*^+/+^ mice (**G**). EE rescued this phenotype by decreasing vGluT1 levels in EE-*Pten*^+/−^ mice, but also elevated vGluT1 in EE-*Pten*^+/+^ mice (**G**). Inhibitory pre-synaptic protein vGAT was reduced in Std-*Pten*^+/−^ mice compared to Std-*Pten*^+/+^ mice, and this phenotype was suppressed by EE (**H**). (**I**,**J**) Synaptosomal levels of excitatory (**I**) and inhibitory (**J**) post-synaptic proteins in the frontal cerebral cortex were largely unaffected by genotype or condition. Excitatory post-synaptic protein PSD-95 trended to increasing in EE-*Pten*^+/+^ mice relative to Std-*Pten*^+/+^ mice (**I**), but no other group differences were found for PSD-95 (**I**) or inhibitory post-synaptic protein gephyrin (**J**). Mean ± SEM. *** *p* < 0.001, ** *p* < 0.01, * *p* < 0.05, ^+^ *p* < 0.10. Std, standard housing; EE, environmental enrichment.

The strongest, most reliable, and most robust neuroanatomical phenotype we have found in the *Pten*^+/−^ mouse model is increased brain size (mass and volume), typically 15–20% in adults on average (e.g., [14,17,19,20]). Our Std-*Pten*^+/−^ showed this characteristic brain mass increase (24% in females, 21% in males), as did the EE-*Pten*^+/−^ mice (20% in females, 22% in males; see Figure 5A,B, Supplemental Table S3). In this cohort, *Pten*^+/−^ mice of both sexes were heavier than their *Pten*^+/+^ littermates. This was largely unaffected by EE, although the EE-*Pten*^+/+^ males showed a trend to decreased body mass relative to Std-*Pten*^+/+^ mice (see Figure 5C,D, Supplemental Table S3). Additionally, this increase in body mass did not account for the increased brain size, as the *Pten*^+/−^ brain enlargement was significant even when normalized to body mass (brain mass/body mass × 100; see Figure 5E,F, Supplemental Table S3). This body weight difference is highly unusual, and has not been found in several other studies to date (e.g., [14,17,19,20,21]). 

We have previously found indications of increased excitatory pre-synaptic protein vGluT1 in the cerebral cortex of mice with conditional haploinsufficiency under the *Emx1-Cre* promotor using histology [22], but levels of cortical excitatory and inhibitory pre- and post-synaptic proteins have not been examined in the germline *Pten*^+/−^ mice. When we compared Std-*Pten*^+/−^ and Std-*Pten*^+/+^ frontal cerebral cortices via western blot using a synaptosomal preparation, we found that not only was vGluT1 elevated in Std-*Pten*^+/−^ mice, but also that inhibitory pre-synaptic protein vGAT was decreased (see Figure 5G,H, Supplemental Table S3). These effects were partially reversed by EE, as there were no significant differences between genotypes in the EE mice for the pre-synaptic proteins (see Figure 5G,H, Supplemental Table S3). These effects were more pronounced for vGluT1: in *Pten*^+/−^ mice, there was a trend to lower protein levels in the EE than Std mice, while in the *Pten*^+/+^ mice, there was a trend to increased vGluT1 with EE (see Figure 5G,H, Supplemental Table S3). We found minimal effects of genotype or EE in the post-synaptic excitatory (PSD-95) or inhibitory (gephyrin) proteins; the only difference was a trend to increased PSD-95 in the EE-*Pten*^+/+^ mice relative to Std-*Pten*^+/+^ mice (see Figure 5I,J, Supplemental Table S3).

These data indicate that while EE does not appear to affect brain mass, it does at least partially reverse the altered excitatory/inhibitory balance seen in the pre-synaptic proteins of Std-*Pten*^+/−^ mice. 

## 4. Discussion

The two main diagnostic criteria for ASD are impaired social behavior and communication, and restricted, repetitive behavior and interests [18], both of which are modeled in *Pten*^+/−^ mice. *Pten*^+/−^ mice show sex-specific social behavior deficits, with females failing to show a preference for the social chamber in the three-chamber social approach test, and males having impaired social recognition on a habituation/dishabituation assay (e.g., [14,17,21,43]). These phenotypes were confirmed in our standard-housed animals and rescued by EE. Similarly, we have previously found that male *Pten*^+/−^ mice dig more and bury more marbles [14,15], indicating an increase in stereotypic, repetitive behavior [42]. We replicated this finding and found that EE partially rescued the increased marble burying increase. 

While we replicated our typical sex-specific social impairments, we also found that *Pten*^+/−^ females had “male-typical” social recognition deficits. Similarly, a previous cohort of *Pten*^+/−^ males has shown a “female-typical” lack of social preference in the three-chamber social approach test [14]. In the current study, we did not find decreased anxiety, as measured by the OFT, in the *Pten*^+/−^ male mice, unlike our previous results [14]. This highlights the importance of testing both sexes and choosing phenotypes that are reliable and replicable, such as the lack of social preference in the three-chamber social approach assay in *Pten*^+/−^ females, and impaired social recognition and increased marble burying in *Pten*^+/−^ males. This also emphasizes that the inclusion of standard or untreated control groups is essential to ensure that manipulations are acting upon the expected baseline and causing the observed effect.

Additionally, we found some unexpected results. The EE-*Pten*^+/+^ males failed to show a social preference in the three-chamber social approach test. While some studies have previously found increased social preferences in enriched male control mice, these typically do not include social enrichment (i.e., more animals per cage than standard-housed mice; e.g., [44,45]). While we do not understand the underlying cause of this behavioral change, we speculate that this lack of social preference may be due to decreased social interest because of the large number of cagemates, including mice from different litters, and/or to increased social avoidance because of increased aggression and dominance hierarchy instability, which have previously been observed in EE-housed male mice [44,46,47,48]. This may also account for the lack of dishabituation in the EE-*Pten*^+/+^ males.

We also found that EE had opposite effects on locomotion in the three-chamber social approach and open field tests. While it is unclear why EE would differentially affect locomotion on these assays, one possibility is that the EE mice explored the open field less because of its similarity to the “play arenas”, thus decreasing the novelty of the environment (both the open field and the play arena apparatuses were white acrylic boxes of similar dimensions, although the play arena time was under red light and included access to several toys, whereas the open field test was empty and under bright white light). Additionally, several studies have found decreased locomotion in the open field in enriched mice, and thus this result is not, in itself, surprising (e.g., [49,50,51,52]). However, it is interesting to note that the two groups that showed increased locomotion in the three-chamber social approach assay were also the two groups that showed an effect of EE on social preference, albeit in different directions. Thus, it is possible that these mice (EE-*Pten*^+/+^ males and EE-*Pten*^+/−^ females) found the stimuli more arousing or were more motivated to approach (females) or avoid (males) the social stimuli than their standard-housed controls.

There is currently no approved pharmacological treatment for the core symptoms of ASD, and early behavioral interventions of various types appear to be the most effective therapeutic option for ASD, with efficacy increasing with earlier ages of commencement [29,30]. However, these are somewhat limited by the fact that most ASD cases are not diagnosed until 2–3 years of age or older, by which it is likely that any neuroanatomical abnormalities will already be established. While the methods of determining equivalent ages in mice and humans lead to inconsistent results, most studies agree that weaning (postnatal day 21, P21) corresponds to at least 2–3 years of age (e.g., [53,54]). Furthermore, there is abundant evidence that brain overgrowth, hyperconnectivity, hyperplasia, hypertrophy, and other neuroanatomical alterations have occurred by this age in *Pten*^+/−^ mice, which are an excellent model for early brain overgrowth [19,20,21]. Thus, we chose to begin EE at P21, after the neuroanatomical abnormalities are established, but before adulthood or the emergence of social behavioral deficits [19,20,33]. In order to maximize the likelihood of an effective intervention, we incorporated all the typical aspects of EE, including enrichment for physical activity (running wheel, larger cage, play arena time), social activity (twice as many cagemates as standard), and sensory activity (larger environment to explore, novel toys, play arena time) [33]. That this complex, multi-modal intervention was able to rescue the most robust behavioral phenotypes of the *Pten*^+/−^ mouse model is exciting and somewhat remarkable. It is likely that the germline haploinsufficiency for *Pten* is affecting multiple systems to enact the different behavioral phenotypes in this model. While there were some unexpected results (e.g., social behavior in EE-*Pten*^+/+^ males), it is not surprising that EE would differentially affect different sexes and different genotypes, especially because of the widespread sexual dimorphism in the *Pten*^+/−^ model, and because of the behavioral differences between *Pten*^+/+^ and *Pten*^+/−^ mice in standard housing conditions. 

However, while this timing, and the fact that we rescued behavior without correcting the gross anatomical abnormality of macrocephaly in these mice, is a promising first step toward the idea of improving outcomes even when diagnosed after the damage is done, there are some limitations to this study that require addressing. First, because we intentionally engaged several modalities in order to increase the chance of successful intervention, we are unable to dissect out the relative contribution of the sensory, social, and physical/exercise aspects of enrichment. Furthermore, particularly due to the multi-modal enrichment we employed, it is difficult to definitively ascertain the mechanism(s) through which EE rescued the behavioral phenotypes. One likely possibility is that EE enhanced synaptic plasticity; this is supported by our data showing that EE corrected the altered pre-synaptic proteins vGluT1 (excitatory) and vGAT (inhibitory) in the *Pten*^+/−^ frontal cortex, thus restoring the excitatory/inhibitory (E/I) balance. However, it should be noted that these data are exclusively from female mice, and confirmation of this result in males is an important future step. It has been frequently hypothesized that E/I imbalance may be a causative or contributing mechanism for ASD by leading to altered connectivity and pathological behavior, and an altered E/I balance has been found in numerous mouse models of ASD (reviewed in [55]). Similarly, other mouse models of ASD have shown altered synaptic plasticity when EE was used to rescue phenotypes (i.e., *Mecp2* and *Fmr1* knockout mice [31,32]). The *Mecp2* knockout study also found increased brain-derived neurotrophic factor levels, which are another of several molecular or cellular mechanisms through which EE could have rescued the *Pten*^+/−^ behavioral deficits [31,34,35,36,37]. Isolating the elements of EE that were effective in rescuing social and repetitive behavior in *Pten*^+/−^ mice, and the mechanisms by which they did so, is the target of future investigations. 

## 5. Conclusions

In the current study, we replicated the social and repetitive behavior deficits previously found in the germline *Pten* haploinsufficient mouse model of ASD and demonstrated that they can be partially or fully rescued by environmental enrichment (EE) that begins at weaning. The timing of this successful intervention demonstrates that EE can rescue ASD-like deficits even after the brain is abnormally hard-wired, possibly through adaptive changes in synaptic plasticity. These promising results provide hope for individuals with late ASD diagnoses and lay the groundwork for determining the mechanism by which EE improves behavioral outcomes, thus potentially opening the door to developing new, effective therapeutics for ASD.

## Data Availability

The data presented in this study are available on request from the corresponding author.

## Supplementary Materials

The following are available online at https://www.mdpi.com/article/10.3390/genes12091366/s1, Table S1: Social behavior statistics, Table S2: Non-social behavior statistics, Table S3: Statistics for post-mortem analyses.
