# Supplementary material for: Environmental Enrichment Rescues Social Behavioral Deficits and Synaptic Abnormalities in Pten Haploinsufficient Mice
Source: Genes (Basel). 2021 Aug 30;12(9):1366. doi: 10.3390/genes12091366 (PMC8468545; doi:10.3390/genes12091366)
Supplement: Supplementary file 1 [file genes-12-01366-s001.zip › Supplemental Tables/Supplemental Table S2.pdf]

Supplemental Table S2. Non-social behavior statistics. Bold indicates significant effects, italics indicate trends.

**Marble Burying**			
Analysis	Effect	Statistics in Females	Statistics in Males
2 (genotype) x 2 (condition) ANOVA on % marbles 2/3+ buried	genotype	*F*(1,65)<0.01, *p*=0.999	***F*(1,56)=7.51, *p*=0.008**
	condition	*F*(1,65)=0.04, *p*=0.834	*F*(1,56)=0.66, *p*=0.420
	genotype x condition	*F*(1,65)=1.79, *p*=0.186	*F*(1,56)=1.24, *p*=0.270
Genotype *t*-test (% marbles 2/3+ buried)	Standard-housed	*t*(29)=1.02, *p*=0.319	***t*(28)=3.09, *p*=0.004**
	EE-housed	*t*(36)=0.92, *p*=0.365	*t*(28)=1.04, *p*=0.307
Condition *t*-test (% marbles 2/3+ buried)	*Pten*$^{+/+}$	*t*(36)=0.79, *p*=0.436	*t*(28)=0.24, *p*=0.815
	Pten$^{+/-}$	*t*(29)=1.14, *p*=0.264	*t*(28)=1.25, *p*=0.222
**Open Field Test**			
Analysis	Effect	Statistics in Females	Statistics in Males
2 (Genotype) x 2 (Condition) x 2 (Thigmotaxis) ANOVA	genotype	*F*(1,67)=0.56, *p*=0.456	*F*(1,55)=0.48, *p*=0.490
	condition	*F*(1,67)=2.16, *p*=0.146	*F*(1,55)=2.02, *p*=0.161
	thigmotaxis	***F*(1,67)=3094.75, *p*<0.001**	***F*(1,55)=3550.69, *p*<0.001**
	genotype x condition	*F*(1,67)=0.19, *p*=0.663	*F*(1,55)=0.27, *p*=0.608
	genotype x thigmotaxis	*F*(1,67)=1.13, *p*=0.291	*F*(1,55)=0.28, *p*=0.602
	condition x thigmotaxis	*F*(1,67)=1.98, *p*=0.163	*F*(1,55)=0.06, *p*=0.810
	genotype x condition x thigmotaxis	*F*(1,67)=0.52, *p*=0.473	*F*(1,55)=0.01, *p*=0.938
2 (Genotype) x 2 (Condition) ANOVA: % center time	genotype	*F*(1,67)=1.15, *p*=0.288	*F*(1,55)=0.13, *p*=0.719
	condition	*F*(1,67)=1.95, *p*=0.168	*F*(1,55)=0.25, *p*=0.619
	genotype x condition	*F*(1,67)=0.53, *p*=0.471	*F*(1,55)<0.01, *p*=0.976
2 (Genotype) x 2 (Condition) ANOVA: distance traveled	genotype	*F*(1,67)=0.36, *p*=0.549	*F*(1,55)=0.14, *p*=0.714
	condition	***F*(1,67)=14.03, *p*<0.001**	***F*(1,55)=5.84, *p*=0.019**
	genotype x condition	*F*(1,67)=0.04, *p*=0.834	***F*(1,55)=6.52, *p*=0.013**
	Post hocs	n/a	Std, WT vs *Pten*$^{+/-}$: *p*=0.125
		n/a	EE, WT vs *Pten*$^{+/-}$: ***p*=0.045**
		n/a	WT, Std vs EE: *p*=0.924
		n/a	*Pten*$^{+/-}$, Std vs EE: ***p*=0.001**
Genotype *t*-test: Standard-housed	% center time	*t*(30)=0.25, *p*=0.801	*t*(28)=0.30, *p*=0.769
	distance traveled	*t*(30)=0.47, *p*=0.641	*t*(28)=1.46, *p*=0.155
Genotype *t*-test: EE-housed	% center time	*t*(37)=1.26, *p*=0.216	*t*(27)=0.24, *p*=0.816
	distance traveled	*t*(37)=0.35, *p*=0.732	***t*(27)=2.21, *p*=0.036**

Condition *t*-test: *Pten*$^{+/+}$	% center time	*t(37)=1.79, p=0.082*	*t*(27)=0.34, *p*=0.737
	distance traveled	***t*(37)=2.94, *p*=0.006**	*t*(27)=0.11, *p*=0.911
Condition *t*-test: *Pten*$^{+/-}$	% center time	*t*(30)=0.40, *p*=0.691	*t*(28)=0.37, *p*=0.716
	distance traveled	***t*(30)=2.39, *p*=0.023**	***t*(28)=3.15, *p*=0.004**
Paired-sample t-test (% time in center vs thigmotaxis): *Pten*$^{+/+}$	Standard-housed	***t*(16)=38.60, *p*<0.001**	***t*(14)=33.75, *p*<0.001**
	EE-housed	***t*(21)=30.08, *p*<0.001**	***t*(13)=27.44, *p*<0.001**
Paired-sample t-test (% time in center vs thigmotaxis): *Pten*$^{+/-}$	Standard-housed	***t*(14)=24.17, *p*<0.001**	***t*(14)=37.31, *p*<0.001**
	EE-housed	***t*(16)=23.92, *p*<0.001**	***t*(14)=24.90, *p*<0.001**
