# Supplementary material for: Environmental Enrichment Rescues Social Behavioral Deficits and Synaptic Abnormalities in Pten Haploinsufficient Mice
Source: Genes (Basel). 2021 Aug 30;12(9):1366. doi: 10.3390/genes12091366 (PMC8468545; doi:10.3390/genes12091366)
Supplement: Supplementary file 1 [file genes-12-01366-s001.zip › Supplemental Tables/Supplemental Table S3.pdf]

Supplemental Table S3. Statistics for post-mortem analyses. Bold indicates significant effects, italics indicate trends.

**Brain, Body Mass**			
Analysis	Effect	Statistics in Females	Statistics in Males
2 (Genotype) x 2 (Condition) ANOVA: brain mass	genotype	**$F(1,63)=115.34$, $p<0.001$**	**$F(1,53)=466.24$, $p<0.001$**
	condition	$F(1,63)=0.62$, $p=0.435$	$F(1,53)=0.62$, $p=0.436$
	genotype x condition	$F(1,63)=0.87$, $p=0.356$	$F(1,53)=0.87$, $p=0.354$
2 (Genotype) x 2 (Condition) ANOVA: body mass	genotype	**$F(1,63)=15.77$, $p<0.001$**	**$F(1,53)=9.17$, $p=0.004$**
	condition	$F(1,63)=1.46$, $p=0.231$	*$F(1,53)=3.36$, $p=0.072$*
	genotype x condition	$F(1,63)=2.77$, $p=0.101$	$F(1,53)=0.32$, $p=0.577$
2 (Genotype) x 2 (Condition) ANOVA: brain % of body	genotype	**$F(1,63)=32.00$, $p<0.001$**	**$F(1,53)=27.95$, $p<0.001$**
	condition	$F(1,63)=2.21$, $p=0.143$	*$F(1,53)=3.99$, $p=0.051$*
	genotype x condition	$F(1,63)=0.07$, $p=0.798$	$F(1,53)=0.02$, $p=0.891$
Genotype *t*-test: Standard-housed	brain mass	**$t(29)=7.06$, $p<0.001$**	**$t(26)=13.51$, $p<0.001$**
	body mass	**$t(29)=3.41$, $p=0.002$**	$t(26)=1.33$, $p=0.195$
	brain % of body	**$t(29)=3.56$, $p=0.001$**	**$t(26)=3.56$, $p=0.002$**
Genotype *t*-test: EE-housed	brain mass	**$t(34)=8.24$, $p<0.001$**	**$t(28)=16.61$, $p<0.001$**
	body mass	*$t(34)=1.94$, $p=0.061$*	**$t(28)=3.19$, $p=0.004$**
	brain % of body	**$t(34)=4.48$, $p<0.001$**	**$t(28)=3.93$, $p=0.001$**
Condition *t*-test: $Pten^{+/+}$	brain mass	$t(36)=1.22$, $p=0.230$	$t(26)=0.39$, $p=0.702$
	body mass	$t(36)=0.39$, $p=0.702$	*$t(26)=1.74$, $p=0.093$*
	brain % of body	$t(36)=0.97$, $p=0.341$	$t(26)=1.35$, $p=0.188$
Condition *t*-test: $Pten^{+/-}$	brain mass	$t(27)=0.11$, $p=0.916$	$t(28)=1.30$, $p=0.204$
	body mass	$t(27)=1.70$, $p=0.101$	$t(28)=1.00$, $p=0.324$
	brain % of body	$t(27)=1.11$, $p=0.278$	$t(28)=1.48$, $p=0.151$

**Synaptosomal Western Blot**		
Analysis	Effect	Statistics
2 (Genotype) x 2 (Condition) ANOVA: vGluT1/β-actin	genotype	$F(1,29)=0.08$, $p=0.786$
	condition	$F(1,29)=0.02$, $p=0.889$
	genotype x condition	**$F(1,29)=5.83$, $p=0.022$**
	Post hocs	Std, WT vs $Pten^{+/-}$: **$p=0.015$**
		EE, WT vs $Pten^{+/-}$: $p=0.220$
		WT, Std vs EE: $p=0.108$
		$Pten^{+/-}$, Std vs EE: *$p=0.090$*

2 (Genotype) x 2 (Condition) ANOVA: vGAT/β-actin	genotype	$F(1,29)=0.50$, $p=0.486$
	condition	$F(1,29)=2.18$, $p=0.151$
	genotype x condition	$F(1,29)=0.44$, $p=0.513$
2 (Genotype) x 2 (Condition) ANOVA: PSD-95/β-actin	genotype	$F(1,29)=0.10$, $p=0.756$
	condition	$F(1,29)=1.37$, $p=0.251$
	genotype x condition	$F(1,29)=1.88$, $p=0.181$
2 (Genotype) x 2 (Condition) ANOVA: gephyrin/β-actin	genotype	$F(1,29)=0.01$, $p=0.924$
	condition	$F(1,29)=1.88$, $p=0.181$
	genotype x condition	$F(1,29)=0.58$, $p=0.454$
Genotype *t*-test: Standard-housed	vGluT1/β-actin	**$t(23)=2.67$, $p=0.014$**
	vGAT/β-actin	**$t(23)=2.17$, $p=0.041$**
	PSD-95/β-actin	$t(23)=1.67$, $p=0.110$
	gephyrin/β-actin	$t(23)=0.60$, $p=0.554$
Genotype *t*-test: EE-housed	vGluT1/β-actin	$t(8)=1.44$, $p=0.192$
	vGAT/β-actin	$t(8)=0.02$, $p=0.989$
	PSD-95/β-actin	$t(8)=0.57$, $p=0.589$
	gephyrin/β-actin	$t(8)=0.64$, $p=0.546$
Condition *t*-test: $Pten^{+/+}$	vGluT1/β-actin	*$t(15)=1.79$, $p=0.094$*
	vGAT/β-actin	$t(15)=1.46$, $p=0.165$
	PSD-95/β-actin	*$t(15)=1.79$, $p=0.096$*
	gephyrin/β-actin	$t(15)=1.74$, $p=0.105$
Condition *t*-test: $Pten^{+/-}$	vGluT1/β-actin	*$t(15)=1.76$, $p=0.098$*
	vGAT/β-actin	$t(15)=0.60$, $p=0.559$
	PSD-95/β-actin	$t(15)=0.14$, $p=0.890$
	gephyrin/β-actin	$t(15)=0.39$, $p=0.704$
