# Supplementary material for: Environmental Enrichment Rescues Social Behavioral Deficits and Synaptic Abnormalities in Pten Haploinsufficient Mice
Source: Genes (Basel). 2021 Aug 30;12(9):1366. doi: 10.3390/genes12091366 (PMC8468545; doi:10.3390/genes12091366)
Supplement: Supplementary file 1 [file genes-12-01366-s001.zip › Supplemental Tables/Supplemental Table S1.pdf]

Supplemental Table S1. Social behavior statistics. Bold indicates significant effects, italics indicate trends.

**3 Chamber Social Approach**			
Analysis	Effect	Statistics in Females	Statistics in Males
2 (Genotype) x 2 (Condition) x 2 (Chamber) ANOVA (% time in chamber)	genotype	$F(1,62)=0.69$, $p=0.409$	$F(1,56)=2.49$, $p=0.121$
	condition	**$F(1,62)=5.19$, $p=0.026$**	$F(1,56)=0.22$, $p=0.644$
	chamber	**$F(1,62)=31.61$, $p<0.001$**	**$F(1,56)=48.51$, $p<0.001$**
	genotype x condition	**$F(1,62)=6.09$, $p=0.016$**	**$F(1,56)=0.68$, $p=0.012$**
	genotype x chamber	$F(1,62)=2.86$, $p=0.096$	$F(1,56)=2.18$, $p=0.146$
	condition x chamber	$F(1,62)=1.82$, $p=0.182$	**$F(1,56)=5.46$, $p=0.023$**
	genotype x condition x chamber	$F(1,62)<0.01$, $p=0.960$	$F(1,56)=1.73$, $p=0.194$
Paired-sample t-test (% time in mouse+tube chamber vs. empty tube chamber): *Pten*$^{+/+}$	Standard-housed	**$t(13)=3.03$, $p=0.010$**	**$t(14)=3.86$, $p=0.002$**
	EE-housed	**$t(20)=4.37$, $p<0.001$**	$t(14)=0.93$, $p=0.370$
Paired-sample t-test (% time in mouse+tube chamber vs. empty tube chamber): *Pten*$^{+/-}$	Standard-housed	$t(14)=1.28$, $p=0.221$	**$t(14)=4.57$, $p<0.001$**
	EE-housed	**$t(15)=2.88$, $p=0.011$**	**$t(14)=5.08$, $p<0.001$**
Paired-sample t-test (distance to mouse+ tube vs. distance to empty tube): *Pten*$^{+/+}$	Standard-housed	**$t(13)=3.55$, $p=0.004$**	**$t(14)=3.88$, $p=0.002$**
	EE-housed	**$t(20)=4.27$, $p<0.001$**	$t(14)=1.22$, $p=0.243$
Paired-sample t-test (distance to mouse+ tube vs. distance to empty tube): *Pten*$^{+/-}$	Standard-housed	$t(14)=1.51$, $p=0.153$	**$t(14)=4.69$, $p<0.001$**
	EE-housed	**$t(15)=2.97$, $p=0.010$**	**$t(14)=5.28$, $p<0.001$**
2 (condition) x 2 (genotype) ANOVA on Distance Traveled	genotype	$F(1,62)=0.93$, $p=0.339$	$F(1,56)=0.08$, $p=0.782$
	condition	**$F(1,62)=5.31$, $p=0.025$**	**$F(1,56)=18.76$, $p<0.001$**
	genotype x condition	$F(1,62)=1.32$, $p=0.254$	*$F(1,56)=3.20$, $p=0.079$*
Genotype *t*-test (distance traveled)	Standard-housed	$t(27)=0.14$, $p=0.894$	$t(28)=1.01$, $p=0.323$
	EE-housed	$t(35)=1.51$, $p=0.141$	$t(28)=1.56$, $p=0.129$
Condition *t*-test (distance traveled)	*Pten*$^{+/+}$	$t(33)=0.96$, $p=0.344$	**$t(28)=4.75$, $p<0.001$**
	Pten$^{+/-}$	**$t(29)=2.12$, $p=0.043$**	$t(28)=1.66$, $p=0.108$
**Social Recognition**			
Analysis	Effect	Statistics in Females	Statistics in Males
2 (Genotype) x 2 (Condition) x 5 (Trial) ANOVA	genotype	$F(1,47)=0.19$, $p=0.669$	$F(1,43)=1.30$, $p=0.261$
	condition	$F(1,47)=0.67$, $p=0.418$	$F(1,43)=0.35$, $p=0.557$
	trial	**$F(4,188)=17.66$, $p<0.001$**	**$F(4,172)=16.34$, $p<0.001$**
	genotype x condition	$F(1,47)=0.85$, $p=0.362$	$F(1,43)=1.32$, $p=0.258$
	genotype x trial	$F(4,188)=0.79$, $p=0.534$	$F(4,172)=0.32$, $p=0.867$
	condition x trial	$F(4,188)=1.06$, $p=0.377$	$F(4,172)=0.33$, $p=0.856$
	genotype x condition x trial	$F(4,188)=1.80$, $p=0.130$	$F(4,172)=0.95$, $p=0.437$

2 (Genotype) x 2 (Condition) ANOVA: H1	genotype	$F(1,47)=1.35$, $p=0.252$	$F(1,43)=0.31$, $p=0.582$
	condition	$F(1,47)=0.28$, $p=0.601$	$F(1,43)=0.03$, $p=0.866$
	genotype x condition	**$F(1,47)=4.38$, $p=0.042$**	*$F(1,43)=3.74$, $p=0.060$*
	Post hocs	Std, WT vs $Pten^{+/-}$: $p=0.471$	Std, WT vs $Pten^{+/-}$: $p=0.155$
		EE, WT vs $Pten^{+/-}$: **$p=0.039$**	EE, WT vs $Pten^{+/-}$: $p=0.606$
		WT, Std vs EE: *$p=0.052$*	WT, Std vs EE: $p=0.494$
		$Pten^{+/-}$, Std vs EE: $p=0.305$	$Pten^{+/-}$, Std vs EE: $p=0.838$
2 (Genotype) x 2 (Condition) ANOVA: Test	genotype	$F(1,47)=0.05$, $p=0.830$	$F(1,43)=1.87$, $p=0.179$
	condition	$F(1,47)=0.92$, $p=0.343$	$F(1,43)=0.09$, $p=0.772$
	genotype x condition	$F(1,47)=0.93$, $p=0.340$	$F(1,43)=0.37$, $p=0.548$
2 (Genotype) x 2 (Condition) ANOVA: Habituation	genotype	*$F(1,47)=2.83$, $p=0.099$*	$F(1,43)=0.30$, $p=0.586$
	condition	$F(1,47)<0.01$, $p=0.983$	$F(1,43)=0.28$, $p=0.601$
	genotype x condition	$F(1,47)=2.36$, $p=0.131$	$F(1,43)=0.25$, $p=0.620$
2 (Genotype) x 2 (Condition) ANOVA: Dishabituation	genotype	$F(1,47)=0.95$, $p=0.334$	$F(1,43)<0.01$, $p=0.962$
	condition	$F(1,47)=0.31$, $p=0.580$	$F(1,43)=0.09$, $p=0.771$
	genotype x condition	$F(1,47)=1.03$, $p=0.314$	**$F(1,43)=5.07$, $p=0.030$**
	Post hocs	n/a	Std, WT vs $Pten^{+/-}$: $p=0.113$
		n/a	EE, WT vs $Pten^{+/-}$: $p=0.124$
		n/a	WT, Std vs EE: *$p=0.057$*
		n/a	$Pten^{+/-}$, Std vs EE: $p=0.204$
Trial ANOVA (H1-H4): Standard-housed $Pten^{+/+}$	trial	**$F(3,48)=5.83$, $p=0.002$**	**$F(3,42)=9.44$, $p<0.001$**
	Post hocs	Hab 1 vs Hab 2: $p=0.529$<br>Hab 1 vs Hab 3: *$p=0.057$*<br>Hab 1 vs Hab 4: **$p=0.003$**<br>Hab 2 vs Hab 3: $p=0.971$<br>Hab 2 vs Hab 4: *$p=0.080$*<br>Hab 3 vs Hab 4: $p=0.721$	Hab 1 vs Hab 2: $p=0.443$<br>Hab 1 vs Hab 3: **$p=0.016$**<br>Hab 1 vs Hab 4: **$p=0.031$**<br>Hab 2 vs Hab 3: **$p=0.016$**<br>Hab 2 vs Hab 4: **$p=0.043$**<br>Hab 3 vs Hab 4: $p=0.999$
Trial ANOVA (H1-H4): EE-housed $Pten^{+/+}$	trial	**$F(3,33)=20.01$, $p<0.001$**	**$F(3,36)=8.08$, $p<0.001$**
	Post hocs	Hab 1 vs Hab 2: $p=0.497$<br>Hab 1 vs Hab 3: **$p<0.001$**<br>Hab 1 vs Hab 4: **$p<0.001$**<br>Hab 2 vs Hab 3: **$p=0.027$**<br>Hab 2 vs Hab 4: **$p=0.013$**<br>Hab 3 vs Hab 4: $p=0.838$	Hab 1 vs Hab 2: $p=0.998$<br>Hab 1 vs Hab 3: **$p=0.009$**<br>Hab 1 vs Hab 4: **$p=0.042$**<br>Hab 2 vs Hab 3: **$p=0.009$**<br>Hab 2 vs Hab 4: *$p=0.075$*<br>Hab 3 vs Hab 4: $p=1.000$

Trial ANOVA (H1-H4): Standard-housed *Pten*$^{+/-}$	trial	**$F(3,36)=6.17$, $p=0.002$**	**$F(3,33)=4.63$, $p=0.008$**
	Post hocs	Hab 1 vs Hab 2: *$p=0.097$*<br>Hab 1 vs Hab 3: $p=0.126$<br>Hab 1 vs Hab 4: **$p=0.011$**<br>Hab 2 vs Hab 3: $p=0.996$<br>Hab 2 vs Hab 4: $p=0.311$<br>Hab 3 vs Hab 4: $p=0.765$	Hab 1 vs Hab 2: $p=0.956$<br>Hab 1 vs Hab 3: *$p=0.086$*<br>Hab 1 vs Hab 4: $p=0.205$<br>Hab 2 vs Hab 3: $p=0.138$<br>Hab 2 vs Hab 4: $p=0.129$<br>Hab 3 vs Hab 4: $p=1.000$
Trial ANOVA (H1-H4): EE-housed *Pten*$^{+/-}$	trial	$F(3,27)=1.73$, $p=0.186$	**$F(3,24)=5.49$, $p=0.005$**
	Post hocs	n/a<br>n/a<br>n/a<br>n/a<br>n/a<br>n/a	Hab 1 vs Hab 2: $p=1.000$<br>Hab 1 vs Hab 3: $p=0.113$<br>Hab 1 vs Hab 4: $p=0.129$<br>Hab 2 vs Hab 3: $p=0.222$<br>Hab 2 vs Hab 4: **$p=0.021$**<br>Hab 3 vs Hab 4: $p=0.889$
Genotype *t*-test: Standard-housed	H1	$t(28)=0.64$, $p=0.529$	$t(25)=1.24$, $p=0.228$
	Test	$t(28)=0.83$, $p=0.415$	$t(23)=1.66$, $p=0.111$
	Habituation	$t(28)=0.11$, $p=0.913$	$t(25)=0.63$, $p=0.532$
	Dishabituation	*$t(28)=1.78$, $p=0.087$*	$t(23)=1.36$, $p=0.188$
Genotype *t*-test: EE-housed	H1	$t(23)=1.36$, $p=0.186$	$t(20)=0.70$, $p=0.494$
	Test	$t(21)=0.42$, $p=0.680$	$t(20)=1.08$, $p=0.292$
	Habituation	$t(23)=1.62$, $p=0.119$	$t(20)=0.05$, $p=0.964$
	Dishabituation	$t(21)=0.02$, $p=0.983$	**$t(20)=2.18$, $p=0.041$**
Condition *t*-test: *Pten*$^{+/+}$	H1	**$t(27)=2.34$, $p=0.027$**	$t(26)=0.70$, $p=0.490$
	Test	$t(27)<0.01$, $p=0.997$	$t(25)=1.23$, $p=0.231$
	Habituation	$t(27)=1.24$, $p=0.224$	$t(26)=0.90$, $p=0.376$
	Dishabituation	$t(27)=0.38$, $p=0.706$	$t(25)=1.67$, $p=0.108$
Condition *t*-test: *Pten*$^{+/-}$	H1	$t(24)=0.06$, $p=0.953$	$t(19)=0.11$, $p=0.913$
	Test	$t(22)=1.41$, $p=0.173$	$t(18)=1.66$, $p=0.114$
	Habituation	$t(24)=0.48$, $p=0.638$	$t(19)=0.19$, $p=0.850$
	Dishabituation	$t(22)=1.04$, $p=0.310$	*$t(18)=1.85$, $p=0.080$*
One-sample t-test (habituation vs. 0): *Pten*$^{+/+}$	Standard-housed	**$t(16)=4.38$, $p<0.001$**	**$t(14)=3.30$, $p=0.005$**
	EE-housed	**$t(11)=7.17$, $p<0.001$**	**$t(12)=3.24$, $p=0.007$**
One-sample t-test (habituation vs. 0): *Pten*$^{+/-}$	Standard-housed	**$t(12)=3.98$, $p=0.002$**	**$t(11)=2.36$, $p=0.038$**
	EE-housed	**$t(12)=2.37$, $p=0.035$**	**$t(8)=2.81$, $p=0.023$**

One-sample *t*-test (dishabituation vs. 0): *Pten*$^{+/+}$	Standard-housed	***t*(16)=3.65, *p*=0.002**	*t(13)=1.87, p=0.084*
	EE-housed	***t*(11)=2.57, *p*=0.026**	*t*(12)=0.23, *p*=0.821
One-sample *t*-test (dishabituation vs. 0): *Pten*$^{+/-}$	Standard-housed	*t*(12)=1.07, *p*=0.305	*t*(10)=0.18, *p*=0.862
	EE-housed	*t*(10)=1.77, *p*=0.107	***t*(8)=4.40, *p*=0.002**
